# Rate discrimination, gap detection and ranking of temporal pitch in cochlear implant users

**DOI:** 10.1007/s10162-016-0569-5

**Published:** 2016-04-21

**Authors:** Stefano Cosentino, Robert P. Carlyon, John M. Deeks, Wendy Parkinson, Julie A. Bierer

**Affiliations:** MRC Cognition and Brain Sciences Unit, 15 Chaucer Rd, Cambridge, CB2 7EF UK; Department of Speech and Hearing Sciences, University of Washington, 1417 NE 42nd St, Seattle, WA 98105 USA

**Keywords:** cochlear implant, rate discrimination, pitch, interleaved procedure, gap detection

## Abstract

Cochlear implant (CI) users have poor temporal pitch perception, as revealed by two key outcomes of rate discrimination tests: (i) rate discrimination thresholds (RDTs) are typically larger than the corresponding frequency difference limen for pure tones in normal hearing listeners, and (ii) above a few hundred pulses per second (i.e. the “upper limit” of pitch), CI users cannot discriminate further increases in pulse rate. Both RDTs at low rates and the upper limit of pitch vary across listeners and across electrodes in a given listener. Here, we compare across-electrode and across-subject variation in these two measures with the variation in performance on another temporal processing task, gap detection, in order to explore the limitations of temporal processing in CI users. RDTs were obtained for 4–5 electrodes in each of 10 Advanced Bionics CI users using two interleaved adaptive tracks, corresponding to standard rates of 100 and 400 pps. Gap detection was measured using the adaptive procedure and stimuli described by Bierer et al. (JARO 16:273-284, 2015), and for the same electrodes and listeners as for the rate discrimination measures. Pitch ranking was also performed using a mid-point comparison technique. There was a marginal across-electrode correlation between gap detection and rate discrimination at 400 pps, but neither measure correlated with rate discrimination at 100 pps. Similarly, there was a highly significant across-subject correlation between gap detection and rate discrimination at 400, but not 100 pps, and these two correlations differed significantly from each other. Estimates of low-rate sensitivity and of the upper limit of pitch, obtained from the pitch ranking experiment, correlated well with rate discrimination for the 100- and 400-pps standards, respectively. The results are consistent with the upper limit of rate discrimination sharing a common basis with gap detection. There was no evidence that this limitation also applied to rate discrimination at lower rates.

## **INTRODUCTION**

A number of studies have identified an association between poor transmission of information by a subset of electrodes and degraded speech perception by cochlear implant (CI) users (Pfingst and Xu 2004; Bierer 2007; Garadat et al. 2012; Long et al. 2014; Noble et al. 2014; Bierer et al. 2015). One approach has been to use psychophysical measures to infer electrode-specific information, such as the relative position of a stimulating electrode to auditory neurons, or the density of healthy neurons responding to that electrode. These measures of the “electrode-to-neuron interface” may then be used to predict performance on everyday listening tasks such as speech reception (Garadat et al. 2012; DeVries et al. 2015). In the present study, three measures of temporal processing were obtained from ten CI users. The aims were to assess the extent of across-electrode and across-subject variation for the different tasks and to identify common processing limitations.

One task is the detection of gaps in continuous signals. For normal hearing (NH) listeners, the detection of short gaps has been modelled as the detection of dips in the output of a sliding temporal window, having an equivalent rectangular duration of about 10 ms (Plack and Moore 1990). Gap detection thresholds (GDTs) may be influenced both by the duration of the window and by the smallest dip that can be detected; this latter factor may in turn depend on the neural representation within that window. Previous studies (Hochmair-Desoyer et al. 1983; Garadat and Pfingst 2011; Bierer et al. 2015) have revealed large across- and within-subject variability in GDTs, suggesting variation in temporal processing along the tonotopic array. Bierer et al. (2015) showed that, although GDTs correlated significantly across electrodes between stimulation in monopolar and partial-tripolar mode, GDTs did not correlate with detection thresholds in either mode. The authors concluded that GDTs revealed a limitation that was separate from, or additional to, that revealed by detection thresholds. Part of the data for the gap detection task analysed here were taken from the study by Bierer et al. (2015).

We compared both the across-electrode and across-subject variation in GDTs with that observed in a rate discrimination task. Normal hearing listeners can detect very small (<1 %) differences in the frequency of low-frequency sinusoids, with difference limens (DLs) increasing gradually above about 2000 Hz and more steeply above about 4000 Hz (Wier et al. 1977). Although there remains some debate concerning the codes that NH listeners use to achieve this exquisite sensitivity, most researchers believe that a temporal code (“phase locking”) is involved for processing frequencies up to 2000 Hz, with many authors suggesting an even higher limit (e.g. Moore and Ernst (2012), but see also Joris and Verschooten (2013)).

In contrast to pure tone discrimination by NH listeners, the detection of pulse rate differences above 300 pps on a single electrode is at chance for the majority of CI users, and even at rates as low as 100 pps, discrimination thresholds are typically greater than the frequency DL for a 100-Hz tone in NH listeners (Shannon 1983; Townshend et al. 1987; Moore and Carlyon 2005; Kong et al. 2009; Carlyon et al. 2010). Research with bandpass-filtered acoustic pulse trains presented to NH listeners have produced results more similar to those obtained with electric pulse trains presented to CIs, although the “upper limit” above which rate discrimination breaks down is about 700–800 pps, which is higher than that observed for the majority of CI users (Carlyon and Deeks 2002; Macherey and Carlyon 2014). Rate discrimination in CI users is likely mediated by phase locking properties of the auditory nerve and brainstem and requires sustained, temporally accurate responses. For discrimination of rates as high as the 400-pps pulse rate used in this study, sustained accurate responses must persist for intervals as short as 2.5 ms, which is close to the typical gap detection thresholds reported by Bierer et al. (2015). Hence, it is possible that performance on these two tasks will correlate more strongly with each other than with rate discrimination relative to the 100-pps standard, which corresponds to a longer (inter-pulse) interval of 10 ms. At this lower rate, the neural response to each pulse may be independent from that to previous pulses, and so performance may be limited by the jitter in the neural response to individual pulses, rather than by the need for temporally accurate firing that is maintained across a higher-rate pulse train.

Understanding the reasons for the limitations in rate discrimination by CI users has clinical as well as scientific relevance: CI companies have developed processing algorithms that represent the signal’s temporal fine structure in the pattern of electrical stimulation, and the success of these algorithms will rest on the ability of CI users to process this temporal information. Here, rate discrimination thresholds were measured for a low-rate (100 pps) and a higher-rate (400 pps) standard. This was done so as to estimate the across-electrode and across-subject variation both in low-rate sensitivity and in the upper limit of rate pitch and to compare these to the corresponding variation in the gap detection task.

Finally, a third task evaluated temporal processing in CI users using a rate pitch ranking procedure (Long et al. 2005). Place pitch ranking—that is, pitch judgments of sounds produced by two different electrodes—is sometimes performed in clinical settings when post-implantation anomalies, such as misplaced electrodes, are suspected (Collins et al. 1997; Kenway et al. 2015). Conversely, rate pitch ranking—which involves pitch judgments of sounds elicited by the same electrode at different stimulation rates—has been explored less extensively (Kong and Carlyon 2010; Macherey et al. 2011). Rate pitch rankings for equal loudness stimuli, and delivered on the same electrode, can provide information about temporal firing activity from a restricted neural population. We measured rate pitch rankings both to identify instances of non-monotonicity (i.e. where an increase in stimulation rate results in a decrease in pitch; cf. Kong and Carlyon (2010)) and to provide an additional check of the robustness of the conclusions obtained from our rate discrimination measures.

## **ACROSS-ELECTRODE AND ACROSS-SUBJECT TASK COMPARISONS**

As noted in the “Introduction”, one aim of the present study was to determine which psychophysical tasks share common limitations, and which do not. The rationale is that, when two tasks are mediated by the same mechanisms and share common limitations, performance will correlate between those two tasks. When evaluating this hypothesis, it is important to differentiate two different sources of variation—that between electrodes in a given subject and that between different subjects.

In a previous study (Bierer et al. 2015), we focussed mainly on across-electrode correlations. There are two advantages in doing so. First, in practical terms, it is possible to re-program an implant so as to avoid “bad” electrodes, and the across-electrode variation in performance on a psychophysical task could inform the choice of which electrodes to de-activate, whereas the across-subject variation could not. Second, unlike across-subject variation, performance differences across electrodes within a given subject cannot be attributed to non-sensory differences between subjects, such as in cognitive skills or the willingness to concentrate on a boring task. As in previous studies (Bierer et al. 2015; Ihlefeld et al. 2015), we evaluated across-electrode variation by subtracting the mean value across electrodes for a given condition within subject. The reduction in degrees of freedom caused by constraining the mean of each subject’s values to be zero was taken into account when measuring statistical significance. The method is equivalent to entering the two measures to be correlated into a univariate ANOVA, with one measure as the dependent variable and the other as the co-variate, and with subjects as a fixed or random factor (Bland and Altman 1986).

A drawback of measuring only the across-electrode correlations is, as noted by Ihlefeld et al. (2015), that this can under-estimate the extent to which two tasks share a common sensory limitation. For example, a likely source of variation in sensory processing arises from differences in neural survival, and these differences may well be greater across subjects than across the auditory nerve array within a single subject. We therefore also report across-subject correlations, calculated from the mean thresholds across all electrodes for each subject, and compare the values of these across-subject correlations between different pairs of tasks. Our rationale is that, if the correlation between two hypothetical tasks A and B is significantly larger than that between tasks A and C, then task A is more likely to share a common basis with task B than with task C. This rationale is valid as long as tasks B and C involve similar cognitive and attentional demands.

## **GAP DETECTION TASK**

### Methods

The majority of the gap detection thresholds (GDTs) analysed here were taken from Bierer et al. (2015). That study obtained data from four or five electrodes in each of nine Advanced Bionics HiRes 90 K CI users. Data were obtained from both ears for one bilateral CI user, thus leading to a total of ten “subjects”. The aim was to compare GDTs to our new rate discrimination measures, and eight of the original ten subjects were available for those tests. We added two new subjects, S48 and C6, who performed both sets of tests, bringing the number of subjects for the gap detection part of the study to a total of 12. The present study involved monopolar stimulation only, but the methods for the two new subjects were in all other respects identical to those described by Bierer et al. (2015). Briefly, listeners performed a two-interval forced-choice gap detection task for a 1031-pps pulse train presented, in different adaptive runs, to one of four or five individual electrodes. Stimuli were presented at the listener’s most comfortable level (MCL). Each symmetric, cathodic-leading biphasic pulse had a phase duration of either 97 or 194 μs, depending on the subject. The nominal duration of the stimuli was 400 ms, roved by ±10 % on each presentation in order to preclude the use of overall duration cues. GDTs were obtained from the average of four or five adaptive runs for each electrode. Signal detection thresholds were also obtained for a 1031-pps 200-ms stimulus for each electrode using the mean of four adaptive runs. The reader is referred to Bierer et al. (2015) for further details. Information about the subjects can be found in Table 1.

**TABLE 1:** Subjects’ details at the time of testing

ID	Age (years)	Deafness onset (age, years)	Possible aetiology	Months of CI use (years)	Took part in experiment
S22	73	55	Hereditary	5.8	1a, 2, 3
S27	84	55–60	Unknown	5.6	1a
S28	75	26	Hereditary	5.4	1a, 2, 3
S30	50	16	Hereditary	10	1a, 2, 3
S39	50	16	Hereditary	30	1a, 2, 3
S48	59	36	Autoimmune disease	2.5	1b, 2
C1	68	32	Unknown	4	1a, 2, 3
C2	32	7	Unknown	3	1a
C3	70	50	Otosclerosis	3	1a, 2, 3
C4	67	37	Otosclerosis	5	1a, 2, 3
C5	54	31	Unknown	5	1a, 2, 3
C6	66	51	Unknown	2	1b, 2, 3

The four experiments were labelled as follows: *1a* – gap detection in Bierer et al. (2015); *1b* – gap detection in this study; 2 and 3 are rate discrimination and pitch ranking in this study. Subjects identified with the letter “C” were implanted and tested in Cambridge, UK; those identified with the letter S were implanted and tested in Seattle, USA

### Results

**FIG. 1:**
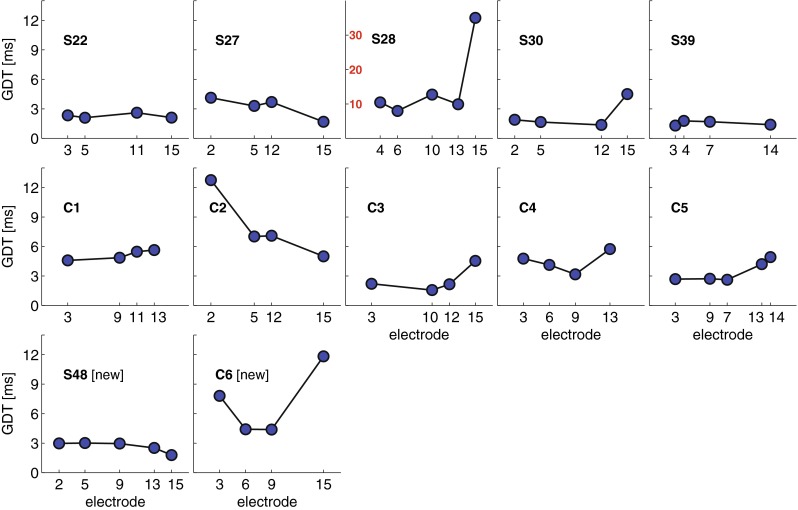
GDTs measured in 12 subjects. Except for subjects S48 and C6, the data are the same as in Fig. 3 of Bierer et al. (2015). Note the different ordinate scale (*in red*) for subject S28.

The results after inclusion of two newly recruited subjects (S48 and C6) are shown in Figure **1**. As was reported for the original ten subjects in Bierer et al. (2015), there was no significant across-electrode correlation between gap detection and signal detection tasks (*r* = 0.24, *p* = 0.1; *df* = 37). Although detection thresholds are not plotted here, for reasons of conciseness, it is worth noting that the very high GDT for S28 on electrode 15 did not correspond to an especially high detection threshold (cf. Fig. 1 in Bierer et al. 2015). This finding suggests that gap detection reveals a source of across electrodes variation that is additional to, or different from, that revealed by detection thresholds. The median of the GDTs for all subjects and electrodes tested was 3.7 ms (median absolute deviation of 2.9 ms).

## **RATE DISCRIMINATION AT 100 AND 400 PPS**

### Rationale

As described in the “Introduction”, one of the aims of the present study was to compare results from the rate discrimination task to those from the gap detection task. A particular prediction was that GDTs would correlate with the upper limit of rate discrimination both across and within subjects. This prediction was based on two observations: First, both tasks may require sustained, temporally accurate firing to high-rate pulse trains, and second, the 300-pps upper limit observed for many CI listeners has a period (3.33 ms) in the range of GDTs observed in the literature and close to the GDTs observed here. Conversely, discrimination of stimuli with longer periods (around 10 ms) may rely on mechanisms that do not require sustained temporally accurate firing to moderate- and high-rate pulse trains.

Four previous methods have been used to measure rate discrimination at high rates. Pitch scaling via magnitude estimation is intuitively appealing but suffers from numerous non-sensory and contextual biases (Poulton 1979). Another method is to measure the next highest rate that listeners can discriminate from a high-rate standard using, for instance, an adaptive procedure (Zeng 2002). A limitation of this method is in the implicit assumption that the upper limit of pitch is lower than the standard rate: For cases where the standard rate is at or above the upper limit, the rate discrimination threshold (RDT) is likely to be unreliable or not measurable. In addition, the use of the same standard on every trial may cause the subject to “overlearn” that stimulus and to use alternative cues (such as small differences in loudness) to identify the signal. To overcome these problems, we have previously used two alternative methods. One is to measure performance as a function of the standard rate with a large (e.g. 35 %) difference between each standard and signal rate and with different standards mixed up within each block of trials (Kong et al. 2009). The other is to use a pitch ranking procedure and to observe the rate above which pitch does not increase (Macherey and Carlyon 2014). Both methods are effective for identifying the upper limit of temporal pitch but, because of the large difference between adjacent pulse rates, may not provide an accurate measure of rate discrimination at low rates. We therefore adopted a new method in which two interleaved adaptive procedures measured RDTs for 100- and 400-pps standards (Jesteadt 1980). In this approach, the signals for the 100-pps standards always had a higher rate than the standard, whilst those for the 400-pps standard had a lower rate. Since for most conditions, a 400-pps standard can be expected to be at or near the upper limit of pitch, the next lowest discriminable rate can be used as an estimate of the upper limit of pitch. We also assume that, even when the subject can detect large differences in rate above 400 pps, there will be some flattening of the pitch ranking function at high rates, and that the RDT will be higher (better) for those electrodes with higher upper limits. This assumption was in fact largely confirmed by the pitch ranking results described in a subsequent section.

### Stimuli and Subjects

Rate discrimination thresholds were measured in eight of the ten subjects from Bierer et al. (2015). Subjects S27 and C2 from the previous study were no longer available and were replaced with subjects S48 and C6. Longer pulse durations were used in Bierer et al. (2015) to prevent reaching compliance when using focussed stimulation modes. However, with our setup and monopolar stimulation, we found that shorter pulse durations allowed finer resolution at high pulse rates. Stimuli were cathodic-phase-leading, symmetric biphasic pulses (43 μs per phase) delivered in monopolar mode. The duration of each signal was 400 ms.

### Loudness Balancing

The different rates were balanced in loudness prior to the rate discrimination task. For each electrode, the approach described by Landsberger and McKay (2005) was used to balance in loudness three rates: 100, 250 and 400 pps. This procedure involved adjusting the level of a 250-pps stimulus to match the loudness of a 100-pps stimulus stimulated at MCL; subsequently, the level of a 400-pps stimulus was adjusted to match the loudness of a 250-pps signal set at the level obtained from the first loudness balancing. Both pairs of balancing runs (i.e. 250 to 100 and 400 to 250) were repeated four times with different starting points, and the results were averaged across runs. After conversion from linear current levels to decibels, a least square fitting was performed on the loudness-balanced levels for the three rates to obtain equal loudness contours for rates from 100 to 400 pps at 1-pps resolution. These levels were used to stimulate at different rates whilst maintaining constant the loudness of the stimuli. When averaged across subjects and electrodes, the balanced MCL for 100 pps was 0.6 and 0.8 dB higher than the MCL for the 250 and 400 pps, respectively. This is consistent with previous evidence that the effect of pulse rate on loudness and threshold is small over the range between 100 and 400 pps (McKay and McDermott 1998).

### Rate Discrimination Procedure

Rate discrimination was measured using two interleaved tracks with a low (100 pps) and a high (400 pps) standard (cf. Jesteadt (1980)). In our interleaved procedure, trials that contained a low or a high rate standard were presented in an intermingled fashion, and the subjects were asked to report the interval that contained the sound higher in pitch. The starting point for both the low-rate and the high-rate tracks was 200 pps. As noted above, the signal was always higher than the 100-pps standard but lower than the 400-pps standard; a response was recorded as correct when the subject identified the higher-rate stimulus as having the higher pitch, and correct answer feedback was provided after every trial. After three consecutive correct responses, the absolute difference between signal and standard rate was reduced by 25 %; this difference was increased by 25 % at every wrong response. The change from increasing to decreasing rate or vice versa was termed a reversal. The test terminated after six reversals had occurred for each track. The likelihood of either the low-rate or high-rate track being selected at each trial (*p*(*L*_100_) or *p*(*L*_400_), respectively) was 0.5 at the start of each run and was thereafter inversely related to the number of reversals completed in each track, rev_100_ and rev_400_, as:

$$ \left\{\begin{array}{c}\hfill p\left({L}_{400}\right)=\frac{N-{\mathrm{rev}}_{400}}{\left(N-{\mathrm{rev}}_{100}\right)+\left(N-{\mathrm{rev}}_{400}\right)}\hfill \\ {}\hfill p\left({L}_{100}\right)=1-p\left({L}_{400}\right)\kern8.25em \hfill \end{array}\right. $$

where *N* = 6 was the total number of reversals; *p*(*L*_400_) was limited in the range [0.2 0.8]. The reversal-dependent switching reduced the time necessary to reach at least six reversals for both tracks. Four interleaved procedures (“runs”) were obtained for each electrode condition, and the results were averaged across runs. Rate discrimination ratios (RDRs) were computed from RDTs as:

$$ \left\{\begin{array}{c}\hfill \mathrm{R}\mathrm{D}\mathrm{R}=\frac{\mathrm{RDT}}{100}\kern1em \mathrm{f}\mathrm{o}\mathrm{r}\ 100\ \mathrm{p}\mathrm{p}\mathrm{s}\ \mathrm{s}\mathrm{tandard}\hfill \\ {}\hfill \kern0.75em \hfill \\ {}\hfill \mathrm{R}\mathrm{D}\mathrm{R}=\frac{400}{\mathrm{RDT}}\kern1em \mathrm{f}\mathrm{o}\mathrm{r}\ 400\ \mathrm{p}\mathrm{p}\mathrm{s}\ \mathrm{s}\mathrm{tandard}\hfill \end{array}\right. $$

### Results

**FIG. 2:**
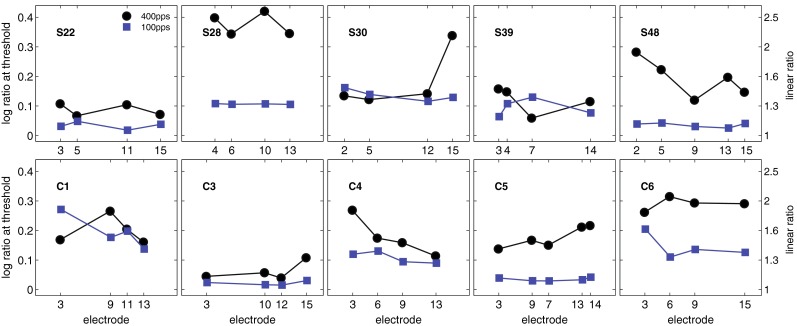
RDRs for ten subjects and for standard rates of 100 pps (*blue squares*) and 400 pps (*black circles*). Data analysis was performed on the logarithms of the RDRs, as shown on the left-hand axis. The raw RDRs are indicated on the right-hand axis. Adaptive tracks for S28 on electrode 15 did not converge to a threshold, and these data points were not included.

RDRs are plotted for the ten subjects in Figure **2**. Overall, moderate across- and within-subject variation was observed. The RDR for S28 on electrode 15 was excluded from the statistical analysis, as the subject failed to converge on a threshold; as discussed later in this manuscript, this was due to a pitch reversal. On average, subjects could discriminate between 100 and 122 pps (equivalent to an average RDR of 1.22) at low rates and between 400 and 268 pps (RDR = 1.49) at high rates. The RDR at 100 pps was higher than the average of 1.07 described by Moore and Carlyon (2005), based on a summary of five different studies, but comparable to the results of a recent study by Stahl et al. (2014). There are multiple possible reasons for the differences in overall performance across studies, including the patient population, the device used and the procedure adopted. For each RDR, both the within- and the across- subject correlations between the first two and last two runs were highly significant (*r* > 0.71; *p* < 0.01). These values provide an estimate of the variability inherent in each measure and provide a useful comparison when interpreting between-measure correlations.

## **RATE PITCH RANKING**

### Rationale

Rate pitch rankings were used as additional measures of temporal processing. Compared to RDRs, results from pitch ranking can demonstrate the presence of pitch reversals, as will be shown to be the case for subject S28, electrode 15. Pitch rankings were also used to provide an alternative estimate of low rate discrimination and of the upper limit of rate discrimination, which we predicted would correlate with RDRs at 100 and 400 pps, respectively. This provided a validation of the rate discrimination measures obtained through the adaptive interleaved procedure. For each electrode, the perceptual pitch ranks of six rates were measured using a mid-point comparison (MPC) technique (Long et al. 2005).

### Stimuli and Subjects

Subjects were asked to make pitch judgments between pairs of 400-ms stimuli presented at one of six possible rates [pps]: 100, 132, 174, 230, 303 and 400. The phase duration of the pulses was 43 μs, and the stimulation mode was monopolar. The levels used for each rate were obtained from the results of the loudness-balancing procedure performed prior to the rate discrimination measures combined with the same interpolation method used for that experiment. The same subjects that took part in the rate discrimination task also completed the pitch ranking task, with the exception of S48 who was no longer available.

### MPC Procedure

The MPC approach is based on a method first described by Steinhaus (1950), and later proposed for measurements of place pitch ranks in brainstem implants (Long et al. 2005). A brief description of the method is as follows. In each run of the MPC, the subject is instructed to select the interval containing the sound higher in pitch in a pair of stimuli. No feedback is provided and the rates of the stimuli in the first pair are picked at random from the six possible options. After the first selection, the interval judged higher is compared with another rate, also selected at random from the remaining four rates, and the subject is again asked to press the button associated with higher pitch. Every new rate to be compared is virtually placed in the middle of the provisional ranking array, followed by a series of comparisons in which the provisional list is bisected; for example, if the new stimulus is judged higher than the middle-ranked stimulus, it is then compared to the stimulus that is mid-way between the middle and highest ranks. The procedure terminates once no more comparisons are possible. The number of comparisons per MPC run is not fixed but, for a set of six stimuli, never exceeds 11. The duration of each run was generally between 30 and 60 s. Ten consecutive MPCs were run per electrode, and the results were averaged across runs.

### Results

**FIG. 3:**
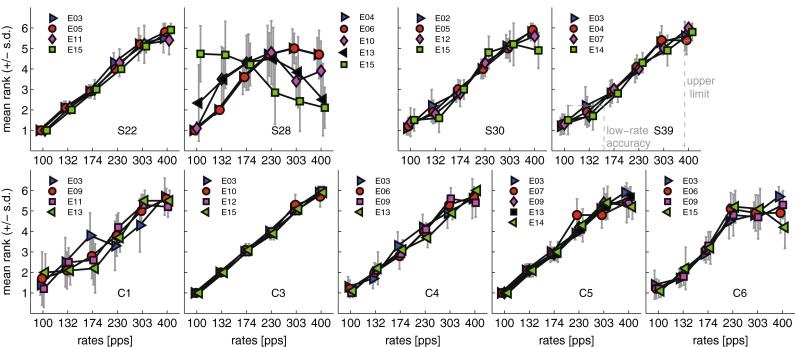
Average pitch ranks as obtained from the MPC procedure. A graphical representation of the method used to compute upper and lower limit of pitch is shown for subject S39 on E14.

The mean pitch ranks for all subjects and electrodes tested are shown in Figure **3** together with standard deviations. Monotonic patterns were observed for most subjects on most electrodes. There were two types of exception to this trend: pitch reversals and a flattening at the higher rates. A marked pitch reversal was observed only for S28 on E15, where the rate at 400 pps was judged lower in pitch than at 100 pps. This is consistent with the inability of this subject to converge on an adaptive threshold for E15 in the rate discrimination experiment, and with the subject reporting that the test often provided the wrong feedback. Additional instances of reversals may be noted at the highest rates for other subject/electrode combinations, such as S28 on E13 and C6 on E15. Occasional temporal pitch ranking reversals have been observed in previous studies, although the reason for them remains unclear (Carlyon et al. 2010; Kong and Carlyon 2010; Macherey et al. 2011). Conversely, flattening of the pitch ranking functions was observed in more subjects, e.g. S30 on E13, C6 on all electrodes, C4 on E9. For these conditions, it is likely that the upper limit of pitch is below 400 pps. In other cases, such as S22 and C3, the functions appear monotonic but the standard deviations are larger at higher rates; generally, the standard deviations for the lowest three rates were significantly smaller than the standard deviation for the higher three rates (*t* test; df = 113, *p* < 0.01). The upper limit of pitch and the accuracy of low-rate encoding (henceforth “low-rate pitch accuracy”) were computed as the rates that produced a pitch rank one standard deviation below the rank for 400 pps or one standard deviation above the rank for 100 pps, respectively (see plot for S39 in Fig. **3** for a graphical representation of the method used). These values were highly correlated with the corresponding RDRs, as described in the next section.

## **DATA ANALYSIS AND DISCUSSION**

### Analysis Methods

Across-task correlations between signal detection, gap detection, rate discrimination (logarithm of the RDRs) and pitch ranking were computed using data from ten CI subjects (or nine for the pitch ranking), as also summarised in Table 1. As confirmed from Figure **3**, S28 reports a pitch reversal on E15. Since adaptive procedures provide unreliable convergence for non-monotonic psychometric functions, the data for S28 on E15 was excluded from the analysis. This caused the correlation between GDTs and RDRs, which were both very large for that subject/electrode combination, to decrease.

### Correlations between rate discrimination and gap detection

**FIG. 4:**
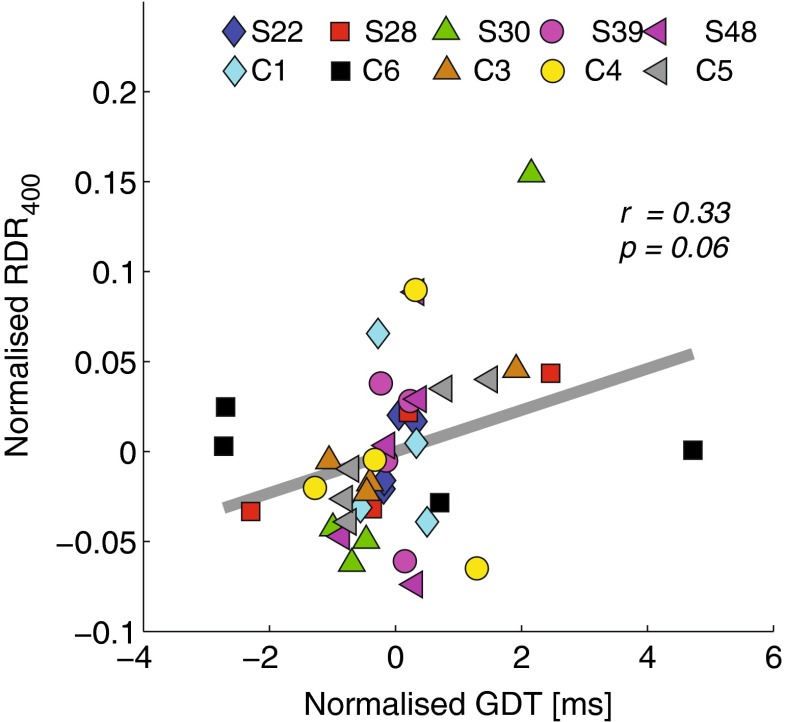
Scatter plot between normalised **RDR**_400_ and **GDT** as measured across electrodes.

No significant across-electrode correlations were observed between RDR_100_ and either the GDT (*r* = 0.03; *df* = 30, *p* = 0.9) nor RDR_400_ (*r* = − 0.12; *df* = 30, *p* = 0.5). A marginally significant correlation was, however, measured between RDR_400_ and GDT (*r* = 0.33; *df* = 30, *p* = 0.06), as shown in the scatter plot of Figure **4**. As discussed below, this general pattern of results was also obtained with the across-subject correlations.

**FIG. 5:**
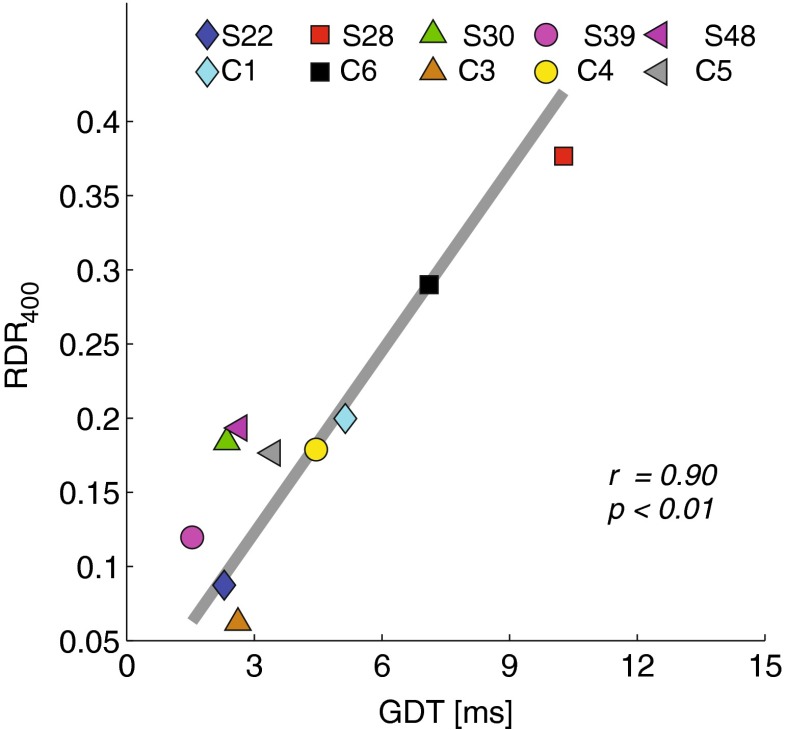
Scatter plot between **RDR**_400_ and **GDT** as measured across subjects.

Across subjects (i.e. averaging over electrodes), the RDR_100_ scores did not correlate with RDR_400_ (*r* = 0.49; df = 8, *p* = 0.15) or with GDT (*r* = 0.43; df = 8, *p* = 0.2). There was, however, a strong and highly significant across-subject correlation between GDT and RDR_400_ (*r* = 0.90; df = 8, p < 0.01), as shown in Figure **5**. Differences in performance across subjects can be broadly attributed to two sources of variance: cognitive and sensory. Cognitive differences are due to, for instance, subject’s concentration, intelligence and to other high level abilities that may affect performance on behavioural tasks. Sensory differences, which may arise from neural survival or from the position of the electrodes inside the cochlea, can also contribute to differences in test performance for different subjects. In our data, the correlation with GDT is significantly greater for RDR_400_ than for RDR_100_ (Williams’ test, *p* < 0.05). Under the assumption that cognitive differences across subjects affect both tasks in equal amounts, this outcome suggests that the across-subject correlation between RDR_400_ and GDT is, at least in part, due to a common sensory limitation, and that the commonality of this limitation is greater than that between RDR_100_ and GDT. It is worth recalling that the two rate discrimination measures were obtained concurrently using two interleaved adaptive procedures, and this may have helped equate cognitive factors between the two tasks. Taken together, the results of both the across-subject and the across-electrode correlations point to a link between gap detection and the upper limit of rate discrimination, but not between the GDT and rate discrimination at 100 pps.

### Relation Between Pitch Ranks and Rate Discrimination Measures

Both the upper limit of pitch and the low-rate pitch accuracy measures correlated with the corresponding rate discrimination measures. The upper limit measured across subjects correlated significantly with RDR_400_ (*r* = 0.89; *df* = 7, *p* < 0.01), and the lower limit correlated with RDR_100_ (*r* = 0.94; *df* = 7, *p* < 0.01). Conversely, RDR_100_ did not correlate significantly with the upper limit (*r* = 0.2; *df* = 7, *p* = 0.6), nor did RDR_400_ correlate with low-rate pitch accuracy (*r* = 0.57; *df* = 7, *p* = 0.1). This argues against the idea that exclusively cognitive factors were responsible for the significant across-subject correlation between the upper limit and RDR_400_, and between low-rate pitch accuracy and RDR_100_.

There was also a significant across-electrode correlation between RDR_400_ and the upper limit of pitch (*r* = 0.52; *df* = 26, *p* < 0.01) after removing across-subject variation. The correlation between RDR_100_ and the lower limit of pitch was not significant (*r* = − 0.13; *df* = 26, *p* = 0.5). However, this lack of significance is perhaps not surprising because, for several subjects, the standard deviation of the pitch rank for the 100-pps stimulus was at—or very close to—zero for all electrodes. Overall, we conclude that the two methods produce consistent measures of the upper limit of pitch and of low-rate pitch accuracy, although the spacing between the two lowest rates was too coarse for an accurate evaluation of across-electrode variations in low-rate pitch accuracy.

### Standard Deviation Ratios Across Measures

For each of the three measures (RDR_100_, RDR_400_ and GDT), a standard deviation ratio (SDR) was computed as the ratio between two values: the *between-electrode* standard deviation, calculated from the standard deviation of the mean GDT s for each electrode, and the *within-electrode* standard deviation, obtained by calculating the standard deviation across adaptive runs for each electrode separately and then averaging these standard deviations across electrodes. As argued by Bierer et al. (2015), the SDR can be used to compare the amount of across-electrode variation between measures that either have different dependent variables, and/or exhibit different sensitivity. The mean SDRs across subjects were as follows: 1.37 ± 0.65 (RDR_400_); 0.83 ± 0.57 (RDR_100_); 2.46 ± 1.62 (GDT). Paired *t* test reported marginally significant differences between SDRs computed from RDR_100_ and RDR_400_ (*df* = 9, *p* = 0.077), and between GDT and RDR_400_ (*df* = 9, *p* = 0.053). A strong statistical difference in SDR was found between GDT and RDR_100_ (*df* = 9, *p* < 0.01). Hence, consistent with other findings in this study, there was a difference between rate discrimination at low rates and both gap detection and, marginally, rate discrimination at high rates. However, the SDR for rate discrimination at high rates was marginally smaller than that for gap detection; this could be due to lower cognitive demands for the gap detection task, which would have reduced the denominator in the SDR (i.e. the *within-electrode* standard deviation). This is plausible because gap detection could be performed by listening for a gap in a single stimulus, which is cognitively less demanding than having the subject to compare two different stimuli, as for rate discrimination. The smaller SDR for rate discrimination at low compared to high rates could be speculated to relate to the neural representation of the pulses at these rates; for instance, an accurate neural representation of every pulse, possible at low rates, would produce a more uniform discriminations across electrodes and subjects than for neural representation that are less locked to the pulse, e.g. at high rates or for the detection of gaps.

### Duration of Deafness and Temporal Processing

Duration of deafness (DoD) prior to implantation was estimated from values reported in Table 1. As reported by Bierer et al. (2015), a statistically significant, positive correlation was observed between DoD and GDT that persisted after the inclusion of subject S48 and C6 from this study (*r* = 0.63; *df* = 10, *p* < 0.05). DoD also correlated with RDR_400_ (*r* = 0.66; *df* = 8, *p* < 0.05), but not with RDR_100_ (*r* = 0.38; *p* = 0.3). However, a re-analysis of the data by Pfingst et al. (1994) by Moore and Carlyon (2005) did find a significant correlation between DoD measured in five subjects and rate DLs at 100 pps. In our study, the correlations with DoD did not differ significantly between RDR_100_ and RDR_400_ (Fisher *z*-transformation, *p* = 0.29).

### The Neural Basis of the Limitations to Rate Discrimination

A number of recent experiments have shed light on the neural basis of the upper limit of rate discrimination. One pertinent finding is that the limitation also applies to tasks that, in bilaterally implanted listeners, involve inter-aural timing judgements, rather than estimates of the pitch of sounds presented to a single ear. Evidence for this comes from two paradigms, one of which investigated whether discrimination between a lower- and a higher-pulse rate, presented to one ear, could be improved by presenting a copy of the lower-rate pulse train to an electrode in the opposite ear, in all intervals of each trial (van Hoesel and Clark 1997; van Hoesel 2007; Carlyon et al. 2008). A benefit occurred when low-rates stimuli were presented contralaterally, thus providing the listener with a binaural cue; the percept of the lower-rate stimulus was reported as fused and was heard in the centre of the head, whereas the higher-rate stimulus was reported to sound diffuse. Conversely, no benefit was observed when a 300-pps stimulus was presented contralaterally to create a binaural cue, showing that limitation in temporal processing, as possibly due to the existence of an “upper limit”, are not restricted to pitch-based tasks. In a second approach, Ihlefeld et al. (2015) measured detection of rate differences as a function of the standard rate for three electrodes in each ear of eight bilaterally implanted listeners. In each ear, the three electrodes were in the base, middle and apex of the array and had a place pitch that was matched to the corresponding electrode in the opposite ear. Ihlefeld et al. (2015) also measured sensitivity to an ITD difference between each matched pair of electrodes as a function of baseline rate. Over the 100–500 pps range studied, both the monaural rate discrimination and the ITD detection became worse at higher rates, also in agreement with previous research (Majdak et al. 2006; Laback et al. 2007; van Hoesel 2007). Importantly, once these general trends were removed, it was possible, to some extent, to predict ITD sensitivity from the worse of the corresponding rate discrimination scores in the two ears. Ihlefeld et al. (2015) concluded that the processing of fine timing differences at high repetition rates is limited by a factor that is not restricted to tasks requiring binaural processing. Interestingly, a study that combined measurements of the electrically evoked compound action potential with rate discrimination tasks, using the same subjects and stimuli, concluded that this limitation lies central to the auditory nerve (Carlyon and Deeks 2015).

The present study adds to this body of knowledge by showing that rate discrimination at high rates is marginally correlated across electrodes, and highly correlated across subjects with another task, gap detection, which involves quite different stimuli. Whereas the rate discrimination and binaural tasks described above used pulse trains having nearly identical rates and levels, the pulse rates in the gap detection stimuli were more than 2.5 times greater than the stimuli in the rate discrimination experiment. Taken together, the results of these studies are consistent with a limitation central to the auditory nerve that is common to tasks that require accurate encoding of short temporal intervals in high-rate stimuli.

An interesting question is why weaker or absent correlations were found between RDR_100_ and either GDTs or RDR_400_. The degradation in temporal processing beyond a certain rate could, in principle, be linked to the existence of a rate-independent temporal jitter in the neural response to each pulse; at high rates, when the jitter period is comparable to the inter-pulse interval, the performance in tasks requiring fine temporal discrimination may deteriorate. A simple model of this type would predict a strong correlation between RDR_100_ and RDR_400_, which in our study was not observed across electrodes or across subjects. A trivial explanation could be that measurements of RDR_100_ were not as reliable as the other measures in this study. However, the test-retest correlation was highly significant, and the RDR measure was sensitive enough to correlate strongly (*r* = 0.89), across subjects, with the low-rate sensitivity obtained from the pitch ranking study. Hence, the absence of a correlation between RDR_100_ and either RDR_400_ or GDT does not seem to be due to inaccuracy in our measurements of low-rate discrimination. Rat her, from the data in this study, it seems low and high rate processing are subject to different sources of limitation, and that the upper limit is due to something specific to the processing of high-rate stimuli rather than to rate-independent jitter. A physiological basis for this limitation is suggested by the finding that neurons in the cat inferior colliculus (IC) produce sustained time-locked responses only at low pulse rates (e.g. Hancock et al. (2012)), with pulse trains having a rate exceeding some limit resulting in only an onset response. Furthermore, the finding that this “upper limit”, as measured in the IC, is influenced by auditory deprivation (Vollmer et al. 2007) is consistent with the correlation between duration of deafness and both RDR_400_ and GDT found in the present study. However, there are at least two reasons for caution when speculating further as to the precise physiological basis for the upper limit. First, although the correlation between deafness duration and RDR_100_ was not significant, it was not significantly smaller than those between deafness duration and either RDR_400_ or GDT. Hence, we do not have strong evidence for a correlation with DoD that is *specific* to high-rate stimuli, and so cannot rule out the possibility of a non-sensory basis for the correlations observed. Second, there is now evidence that the upper limit to which IC responses phase lock is influenced, in animal experiments, by the anaesthesia used to obtain those recordings (Chung et al. 2014). Such limitations will not, of course, apply to the human subjects performing psychophysical tasks.

### Pitch Reversals

Cases of rate pitch reversals have been reported in previous studies (Kong and Carlyon 2010; Macherey et al. 2011; Macherey and Carlyon 2014). A definite instance of pitch reversal was shown also in this study for subject S28 on electrode 15 (cf. Fig. **3**). A practical consideration about pitch reversals concerns the appropriateness of providing feedback in two-interval rate discrimination tasks, as this may affect both the thresholds for the electrode that shows a pitch reversal, and the thresholds measured in other electrode conditions.

In principle, a rate pitch reversal could arise from changes in either a spatial (place-of-excitation) or temporal code. The former could arise if the reduction in current needed to keep loudness constant for different pulse rates had an asymmetric effect on the spatial spread of excitation, so as to bias it more towards the apex or the base. One way in which the temporal code could change in a paradoxical manner is suggested by the fact that, at moderate-to-high pulse rates, the electrically evoked compound action potential (ECAP) is modulated, being larger for odd than for even numbered pulses (Wilson 1997). Carlyon and Deeks (2015) have showed that the depth of this ECAP modulation increases with increasing pulse rate, and thus have suggested that beyond a high pulse rate, the modulation may be so pronounced as to transmit only the odd numbered pulses to the brain. This could in principle counteract, and even reverse, the increase in pitch caused by the increased pulse rate. By comparing rate discrimination and ECAP modulation with the same subjects and stimuli, Carlyon and Deeks (2015) were able to show that this ECAP modulation was not sufficient to explain the upper limit of rate discrimination for their group of subjects. However, it remains possible that, for a minority of subject/electrode combinations, the modulation in the neural response—and, specifically, its increase with increasing pulse rate—is exceptionally large so as to cause pitch reversals. It is also of course possible that, as is believed to be the case for the upper limit of pitch, rate pitch reversals arise from changes in temporal processing at sites central to the auditory nerve (Van Wieringen et al. 2003).

## **SUMMARY OF RESULTS**

(i) There was a highly significant across-subject correlation, and a marginally significant across-electrode correlation, between rate discrimination for a high-rate standard and gap detection. This is consistent with these two tasks sharing similar temporal processing mechanisms, such as those necessary for fast, sustained and temporally accurate firing.
(ii) In contrast, rate discrimination at low rates did not correlate across electrodes either with rate discrimination at high rates or with gap detection thresholds. This is consistent with there being different sources of limitation for rate discrimination at low and at high rates.
(iii) The lower and upper limits estimated from pitch ranks correlated strongly with rate sensitivities at 100 and 400 pps, respectively.
